# A Rare Case of Rivaroxaban Causing Delayed Symptomatic Hepatocellular Injury and Hyperbilirubinemia

**DOI:** 10.1155/2017/5678187

**Published:** 2017-01-30

**Authors:** Keith Glenn, Patrick Chen, Mustafa Musleh, Rao Pallivi, Melissa Grilliot

**Affiliations:** Department of Internal Medicine, Wright State University Boonshoft School of Medicine, Dayton, OH, USA

## Abstract

*Importance*. As Rivaroxaban has increased in popularity, it has been accompanied with a growing body of evidence displaying its ability to cause drug induced liver injury (DILI).* Observation*. A 74-year-old Caucasian female developed Rivaroxaban DILI two weeks after finishing a 14-day course. The patient was symptomatic and jaundiced with elevated transaminases and hyperbilirubinemia with normal lab values two months priorly. Liver biopsies showed mixed inflammatory infiltrate of lymphocytes, neutrophils and eosinophils, rare necrotic hepatocytes, and canalicular and intrahepatocellular cholestasis, all of which are consistent with DILI.* Conclusion and Relevance*. We present this case to add to the growing evidence that Rivaroxaban can be associated with severe, symptomatic liver injury and to ensure physicians are aware of these possible side effects of novel anticoagulants with their increasing use.

## 1. Introduction

Rivaroxaban, a direct factor Xa inhibitor, has gained popularity due to the advantage of daily oral administration without requiring coagulation monitoring. This drug was approved in the United States for treating embolism in patients with nonvalvular atrial fibrillation, for prevention of deep vein thrombosis after orthopedic surgery, and for treating deep vein thrombosis or pulmonary embolism [1, 2]. It is metabolized by CYP450 enzymes and has dual mode of elimination with one-third excreted by the liver and two-thirds by the kidney [3–6].

Common side effects of Rivaroxaban include hemorrhage in the gastrointestinal tract and rarely in the brain or spinal cord. Other side effects are skin reactions and liver injury. Symptomatic liver injury has recently been reported in 17 patients thus far. We report a case of severe, symptomatic Rivaroxaban DILI in a patient that presented 14 days after completing a 14-day course.

## 2. Case

**Figure 1:**
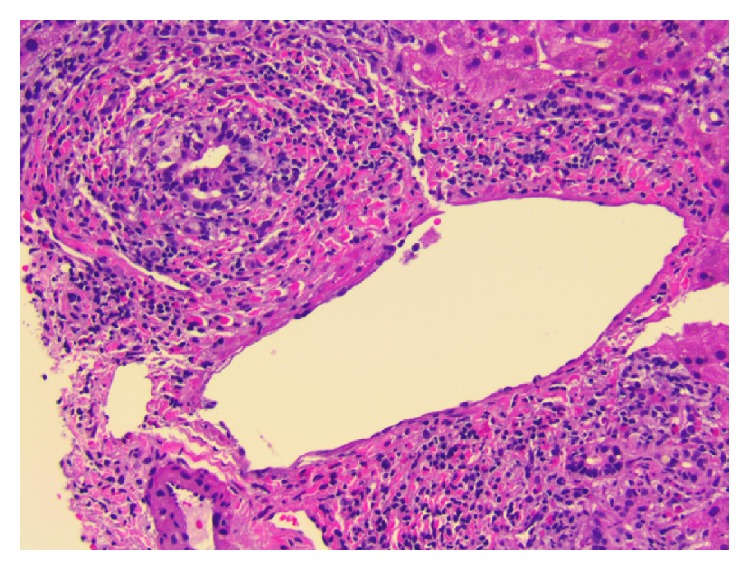
Histological view for biopsy of liver tissue illustrating portal tracts with mixed inflammatory infiltrates composed of lymphocytes, neutrophils, and eosinophils. Hematoxylin/eosin staining 20x.

**Figure 2:**
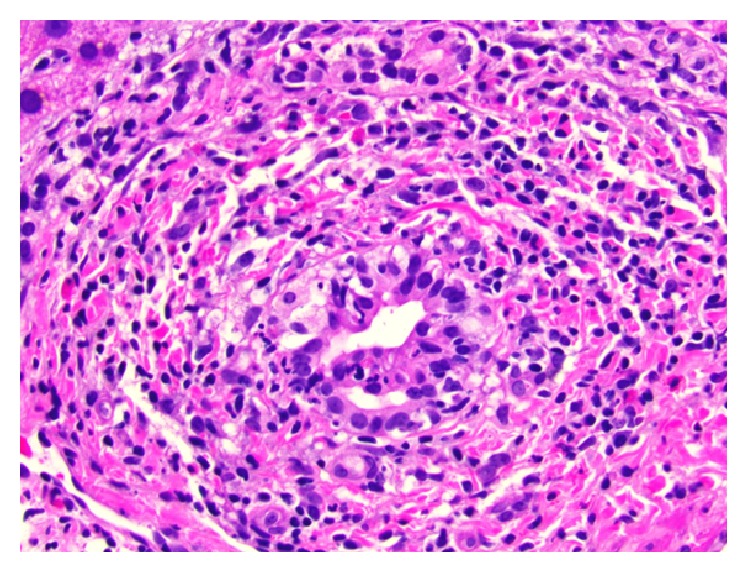
Histological view for biopsy of liver tissue illustrating neutrophils within bile ductular epithelium. Hematoxylin/eosin staining 40x.

A 74-year-old Caucasian female patient presented with profound fatigue and yellowish discoloration of her skin and eyes. She was recovering from a recent uneventful right knee arthroplasty, completed one month prior to presentation. She was discharged home on a 14-day course of Rivaroxaban for postoperative deep venous thrombosis prophylaxis. Additional medications since surgery included 2 tablets of tramadol and 2 tablets of acetaminophen-hydrocodone total in the immediate postoperative period. Physical exam was remarkable for generalized jaundice and icteric sclera. Her labs showed elevated bilirubin up to 7.5 mg/dL (upper limit normal (ULN) 1.2 mg/dL), direct bilirubin 6.7 mg/dL (ULN 0.4 mg/dL), alanine aminotransferase (ALT) 506 u/L (ULN 60 u/L), aspartate aminotransferase (AST) 197 u/L (ULN 55 u/L), and alkaline phosphatase 332 u/L (ULN 144 u/L). Complete blood count was within normal limits and INR was 0.9. Her liver function tests had been normal two months priorly. Extensive workup for other causes of liver injury was negative including viral hepatitis serologies, anti-nuclear antibodies, smooth muscle antibodies, anti-mitochondrial antibodies, ceruloplasmin, acetaminophen level, and iron studies. A right upper quadrant ultrasound with doppler was normal except for evidence of prior cholecystectomy with non-dilated common bile duct. Magnetic resonance cholangiopancreatography (MRCP) was normal. A liver biopsy showed mixed inflammatory infiltrate of lymphocytes, neutrophils, and eosinophils with rare necrotic hepatocytes and canalicular and intrahepatocellular cholestasis, all of which are consistent with DILI (Figures 1 and 2). At her 2-week follow-up enzymes were trending down with resolution of fatigue and jaundice. There was complete resolution at one year.

## 3. Discussion

There have been 17 reported cases of Rivaroxaban DILI per recent systematic review assessing causality done by Björnsson and Hoofnagle, using a recently developed open access website established by the National Institutes of Health called LiverTox (http://livertox.nih.gov) [7]. Rivaroxaban is currently a category B agent with the number of published cases of DILI falling in the range of 12 to 49 with no cases to date published indicating acute liver failure, chronic hepatitis, or vanishing bile duct syndrome [8]. Of the 17 cases reported, all patients recovered upon stopping Rivaroxaban within two to four weeks [8].

This patient showed a delayed presentation of Rivaroxaban DILI with symptoms and presentation starting about two weeks after discontinuation, which is in contrast to the current growing body of literature [8]. Our patient fulfilled Hy's law that states drugs causing hepatocellular injury show 3-fold greater elevations of ULN AST or ALT, total bilirubin greater than two times ULN without initial findings of cholestasis, and no other reason to explain the etiology such as other drugs and viral hepatitis. The clinical picture of prior cases of Rivaroxaban DILI had 11 patients with similar presenting features of decreased appetite, nausea, and jaundice [9]. However, unlike other documented cases the patient presented with symptoms 14 days after the discontinuation of the drug. Because Rivaroxaban has a half-life of ten hours, there is classically noted improvement shortly following drug discontinuation, making our case unique and a more challenging diagnosis [2].

Upon review of prior cases of accepted DILI from Rivaroxaban there were variations in the workup to rule out other etiologies of liver injury secondary to differential diagnosis prompted by clinical presentation and comorbidities [9]. Due to nonspecific testing, biomarkers, or pathognomonic histology, this is a diagnosis of exclusion [10]. The patient's imaging, including US and MRCP, was negative. The biopsy and acetaminophen level were not congruent with acetaminophen toxicity. Other potential causes such as autoimmune hepatitis, sepsis, Wilson's disease, hemochromatosis, and viral hepatitis were ruled out. Liver biopsy showed mixed inflammatory infiltrate with eosinophils and no presence of fibrosis or ductopenia similar to other biopsies reported in two cases by Russmann et al. [9]. Given negative autoimmune serology, no other potential drug causes, eventual full resolution, and temporal relationship, though delayed compared to prior cases, make Rivaroxaban DILI the most likely etiology for this patient's presentation.

## 4. Conclusion

We present this case of Rivaroxaban drug induced liver injury to add to the growing body of evidence and increase physician awareness. Physicians should be cognizant of this rare, but serious, side effect as nonvitamin K anticoagulants are becoming increasingly popular. Further studies are needed to elucidate possible mechanisms for Rivaroxaban DILI and determine those who may be more susceptible. It is important to keep DILI in the differential diagnosis and increase monitoring vigilance, as it could possibly result in acute liver failure, requiring emergent liver transplantation or resulting in death.
